# Predicting diabetes mellitus genes via protein-protein interaction and protein subcellular localization information

**DOI:** 10.1186/s12864-016-2795-y

**Published:** 2016-08-18

**Authors:** Xiwei Tang, Xiaohua Hu, Xuejun Yang, Yetian Fan, Yongfan Li, Wei Hu, Yongzhong Liao, Ming cai Zheng, Wei Peng, Li Gao

**Affiliations:** 1 School of Information Science and Engineering, Hunan First Normal University, Changsha, 410205 China; 2 College of Computing and Informatics, Drexel University, Philadelphia, PA 19104 USA; 3 College of Computer, National University of Defense Technology, Changsha, 410073 China; 4 School of Mathematical Sciences, Dalian University of Technology, Dalian, 116023 China; 5 Computer Center, Kunming University of Science and Technology, Kunming, 650500 China; 6 School of Computer, Central China Normal University, Hubei, 430079 China

## Abstract

**Background:**

Diabetes mellitus characterized by hyperglycemia as a result of insufficient production of or reduced sensitivity to insulin poses a growing threat to the health of people. It is a heterogeneous disorder with multiple etiologies consisting of type 1 diabetes, type 2 diabetes, gestational diabetes and so on. Diabetes-associated protein/gene prediction is a key step to understand the cellular mechanisms related to diabetes mellitus. Compared with experimental methods, computational predictions of candidate proteins/genes are cheaper and more effortless. Protein-protein interaction (PPI) data produced by the high-throughput technology have been used to prioritize candidate disease genes/proteins. However, the false interactions in the PPI data seriously hurt computational methods performance. In order to address that particular question, new methods are developed to identify candidate disease genes/proteins via integrating biological data from other sources.

**Results:**

In this study, a new framework called PDMG is proposed to predict candidate disease genes/proteins. First, the weighted networks are building in terms of the combination of the subcellular localization information and PPI data. To form the weighted networks, the importance of each compartment is evaluated based on the number of interacted proteins in this compartment. This is because the very different roles played by different compartments in cell activities. Besides, some compartments are more important than others. Based on the evaluated compartments, the interactions between proteins are scored and the weighted PPI networks are constructed. Second, the known disease genes are extracted from OMIM database as the seed genes to expand disease-specific networks based on the weighted networks. Third, the weighted values between a protein and its neighbors in the disease-related networks are added together and the sum is as the score of the protein. Last but not least, the proteins are ranked based on descending order of their scores. The candidate proteins in the top are considered to be associated with the diseases and are potential disease-related proteins. Various types of data, such as type 2 diabetes-associated genes, subcellular localizations and protein interactions, are used to test PDMG method.

**Conclusions:**

The results show that the proteins/genes functionally exerting a direct influence over diabetes are consistently placed at the head of the queue. PDMG expands and ranks 445 candidate proteins from the seed set including original 27 type 2 diabetes proteins. Out of the top 27 proteins, 14 proteins are the real type 2 diabetes proteins. The literature extracted from the PubMed database has proved that, out of 13 novel proteins, 8 proteins are associated with diabetes.

## Background

Diabetes mellitus (often also known as diabetes) is a set of metabolic disorders. The latest data from World Health Organization (WHO) (http://www.who.int/diabetes/en/) shows that 9 % of adults worldwide are affected with diabetes. In 2012, 1.5 million people died of the disease. WHO points out that diabetes will become the No. 7 pestilence of threatening the human survival in 2030. It is estimated that America spent $245 billion treating diabetes in 2012 (http://www.diabetes.org/). Among these costs, $176 billion is directly allocated for medical expenditures, while the remaining funding is used for increasing productivity. Therefore, Diabetes mellitus has evoked great concern in the public health.

In diabetes mellitus, blood sugar levels cannot be reasonably adjusted by the body [1]. For a person with diabetes, the pancreas fails to make sufficient insulin, improperly uses the insulin, or both. In the fast-flowing blood, insulin and glucose work together. The former helps the laster to come into cells of the body and produce energy. Sugar is unable to enter the cells if the insulin does not function properly. This results in the amount of glucose in the blood to go steadily up until generating the high concentration of blood sugar, and causing the cells in the lack of fuel.

Typically, diabetes can be classified into three categories: type 1 diabetes, type 2 diabetes (T2D) and gestational diabetes. When beta cells in the pancreas are destroyed and unable to produce, store, and release the hormone insulin, type 1 diabetes(formerly known as insulin-dependent) occurs [2]. In people with type 1 diabetes, the levels of the blood sugar have not been properly controlled due to the deficient insulin production. The patients with type 1 diabetes often have to regularly inject insulin which help to control their blood sugar. In type 2 diabetes (referred to as non-insulin-dependent), beta cells are able to secrete enough insulin but the body cannot use the insulin effectively and attempts to compensate by making a higher quantity of insulin [3], causing insulin resistance. The production of hepatic glucose cannot be suppressed because of hepatic insulin resistance, and the ability to absorb peripheral glucose is impaired by peripheral insulin resistance. The two factors lead to fasting and postprandial hyperglycemia. The report by World Health Organization (WHO) reveals that 90 % of diabetics worldwide have T2D. In the past three decades the number of persons with T2D has increased sharply in countries of all income levels (http://www.who.int/diabetes/en/). Gestational diabetes mellitus is a condition where women without prior history of diabetes develop glucose intolerance and high concentration of blood sugar during pregnancy (usually in the third trimester) [4]. Women who had been attacked by gestational diabetes are more likely to develop type 2 diabetes later in life. Diabetes is caused by various factors. The inherited factors, i.e., genetically determined abnormalities of insulin action play an important role. The scope of metabolic abnormalities related to variations of the insulin receptor may cover hyperinsulinaemia and mildly high blood sugar levels to symptomatic diabetes [5–7]. For example, certain mutations of some genes like HLA-DQA1, HLA-DQB1 and HLA-DRB1 raise the risk of causing type 1 diabetes. A few vital proteins in the immune system are generated according to the instructions from these genes [8–10]. Predicting diabetes-associated proteins is very important to understand how diabetes develops since most diabetes-associated variations have an impact on the function of proteins. Linkage studies are often used to determine the genomic intervals which are linked to the disease of interest [11]. Prioritizing a mass of candidate genes via experimental technologies is so expensive and time-consuming that it becomes often impossible to detect the real disease genes by analyzing the list of genes belonged to the interval. Consequently, computational methods have been becoming a prominent option to address such problems.

A lot of computational methods have been developed to sequence and predict the most likely disease-related genes by combining various types of data from different sources, for instance, gene expressing profiles [12, 13], functional annotation information [13–17] and sequence-based features [18]. Meanwhile, huge amounts of protein-protein interactions produced by high-throughput technologies play an important role in the disease identification since they offer functional information in a network environment [19]. Furthermore, the proteins coded by genes which are linked to a specific or familiar disease phenotype tend to stay together and form clusters in the protein-protein interaction network [20]. In 2006, it was reported that exploiting protein-protein interactions brings prediction of positional candidate disease genes much closer to the possibility. A large scale usage of PPIs can predict novel candidate proteins [21]. Many methods and frameworks based on the protein-protein interaction networks have been proposed to rank or identify potential disease candidate genes to understand genetic diseases. For Alzheimer Disease, a list of candidate genes/proteins are prioritized by a computational method in terms of the public human protein-protein interaction networks (PPINs) [22]. In the paper of Erten et al., the topological similarity in the human PPINs is employed to prioritize candidate disease genes [23]. Nevertheless, using the PPINs is a risky choice since false interactions made by high-throughput experiments have a negative impact on the disease gene prioritization [24–28]. In order to mitigate that particular risk, new methods are developed to identify candidate disease genes or proteins via integrating biological data from other sources. In the work of Wu et al., the gene expressing data are integrated with the PPI data to identify cancer-related genes [29]. The functional similarity of Gene Ontology is combined with protein protein interactions (PPIs) to prioritize candidate cardiomyopathy genes [30]. However, these methods neglect the fact that proteins are unable to conduct the desired functions until they take up the correct subcellular compartments. More specifically, a protein can interact with another one only if they are localized at the same subcellular compartments [31, 32].

In this article, we propose a method, i.e., Predicting Diabetes Mellitus Genes (PDMG), to rank candidate diabetes mellitus genes by incorporating protein subcellular localization information into the protein-protein interaction networks. First, the protein subcellular localization data are incorporate into the PPINs and the weighted networks are built. Second, we collect the gene records of diabetes from Online Mendelian Inheritance in Man (OMIM) and extract seed genes from these records. Only the genes of T2D are retained since the genes of other diabetes subtypes in OMIM are rare. Then T2D-specific PPINs are constructed by utilizing seed proteins and their interacting neighbors (candidate proteins) from the weighted PPINs. Subsequently, we compute the disease-associated score for each protein in the T2D-specific networks and sort them in descending order. Finally, we discuss the top 27 candidate proteins.

## Methods

**Fig. 1:**
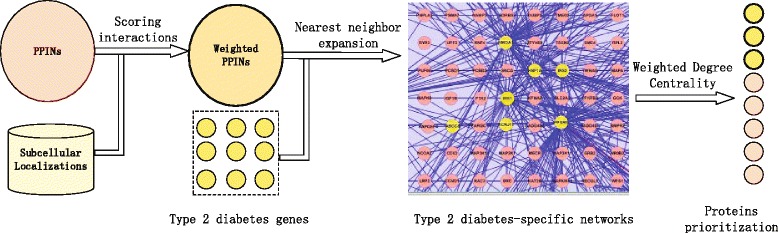
The schematic of PDMG algorithm for sequencing candidate proteins of the disorders. Our method is mainly comprised of three steps, building weighted PPINs, producing T2D-specific networks and prioritizing candidate proteins

In this section, the PDMG method is introduced in detail (see Fig. 1). We first give a general description of sequencing problem of the disease genes. Subsequently, the technology incorporating the subcellular localization information into the PPINs is discussed. Furthermore, we elaborate the method of building disease-specific networks starting from the known disease genes/proteins. Finally, we describe the prioritizing approach of candidate diabetes genes/proteins based on the disease-specific networks.

### Disease gene prioritizing problem

In bioinformatics, the predicting problem of genes that have a close relationship with complex diseases is actually converted into node prioritization problem. The nodes representing the candidate genes/proteins will be scored in accordance with one or more strategies. Then the scores are used to rank them. There is an interesting phenomenon in the biological networks, i.e., the ’guilt-by-association’ principle. It depends on the assumption that the genes/proteins leading to diseases tend to have the similar or same properties [33]. In term of the principle, people can extract a group of disease-causing genes from the disease databases as the original seed proteins and then quantify the associations between the candidate genes and seed genes. Consequently, the candidate genes are sorted according to the associations [13, 34].

Let *D* indicate a disease of interest. *S* is a seed gene set in which the genes are associated with *D*. The candidate protein/gene set, represented *C*, is mechanistically associated with *D*. The sets *S* and *C* constitute the inputs of the disease gene prioritizing algorithm. The known genes in *S* related to *D* serve as a starting point for prioritizing the proteins/genes in *C*. Next, capturing the relationships between the genes in *C* and the genes in *S* becomes a critical step. This need to use the topological characteristics of human PPINs. The PPINs, denoted *G*=(*V*,*E*,*w*), consisting of a group of proteins *V* and undirected interactions *E* among the proteins. Meanwhile, *uv*∈*E* indicates the interaction between *u*∈*V* and *v*∈*V*. Due to the false positive rate of the protein-protein interaction data, it is necessary to assigned a weight value to each interaction *uv*∈*E*. The confidence scores represent the reliability of the interactions between *u* and *v*.

In this article, protein subcellular localization data is used to calculate the confidence scores between proteins. The candidate gene products are sorted based on the scores.

### Scoring PPIs

The eukaryotic cells are elaborately organized into functionally-distinct intracytoplasmic "inclusions" or compartments enclosed within membranes, such as a nucleus and other organelles. The compartments specialize in performing all types of biological functions. The micro-environments have significant influence over protein functions since they control access to and availability of various interacting proteins. In essence, the interactions strongly converge among proteins which are located in the same area of the cells(one-sided binomial test with *P* < 0.001), but the degree of concentration widely depends on the compartments [35]. For instance, the interactions between cytoplasmic proteins are 1.3-fold converged above the threshold. Instead, the interactions among microtubule proteins are 56-fold converged above the threshold. This suggests that the compartment shared by two interacting proteins in the microtubule cytoskeleton better explains the physical and functional interaction than the area of the cytoplasm in which the proteins interact [35]. The fact demonstrates that the significance of different compartments is different in cell activities. After investigating the associations between subcellular localizations and PPIs, Peng et al. find that the farmer is helpful for identifying essential proteins [36, 37]. They give us the motivation of using subcellular localizations to predicting candidate disease genes. Moreover, my research suggests that over half of the T2D genes code essential proteins. Thus, we reason that subcellular localization information can improve the methods of prioritizing candidate disease genes.

Peng et al. report that the significance of a compartment is not out of proportion to the number of interacted proteins in this compartment [36]. In order to score the compartments, the number of the proteins in each compartment is counted. For every compartment, its score is described as the number of interacted proteins in the compartment, denoted by *C*_*X*_, divided by the number of proteins in the largest size compartment (consisting of the largest number of interacted proteins), represented by *C*_*M*_. The score *SC* is calculated by using

1 $$ SC(I)=\frac{C_{X}(I)}{C_{M}},  $$

From the formulation, the value of *SC* ranges from 0 to 1, where *I* ∈ {1, 2, …, 11}.

According to the scores of compartments, the interactions between proteins in the PPINs can be weighted. The different scores of the compartments mean that some compartment are more important than other ones. The phenomenon leads to the importance of PPIs taken place in different compartments should also be different.

Consider a set of compartments *Loc*(*u*) where protein *u* is localized. For the two proteins of an interaction (*u*,*v*), each protein might be annotated by multiple subcellular localizations. It is reasonable that the interacted protein pairs are localized at the same compartment. Therefore, the interaction (*u*,*v*) can be annotated by the shared compartments, i.e., $SLoc(u, v)=Loc(u)\bigcap Loc(v)$. Furthermore, the score of the interaction (*u*,*v*) is defined as

2 $$\begin{array}{@{}rcl@{}} W(u, v)=\left\{ \begin{array}{ll} max(SC(I)),if\ \ SLoc(u, v)\neq \Phi\\ SC(C_{N}), otherwise \end{array} \right. \end{array} $$

If *SLoc*(*u*,*v*)≠*Φ*, the score of the interaction (*u*,*v*) is assigned with the maximum value of score of the shared compartments. Since the subcellular localization information of some proteins may be missing, for the interactions with *SLoc*(*u*,*v*)=*Φ*, the scores of these interactions are assigned with the minimum value of *SC*(*I*) among compartments. In Eq. 2, *C*_*N*_ is the compartment with the smallest size.

### Disease-specific networks

The OMIM database (http://www.omim.org/) severs as the starting point to extract an initial collection of disease-associated genes, i.e., the seed set *S*. With the seed genes and weighted PPIs, we derive a disease-specific networks in terms of the nearest-neighbor expansion approach. In other words, the disease-related networks consist of the seed proteins and their direct neighbors.

### Prioritizing candidate disease gene products

In this subsection, we score the proteins in the disease-specific networks and rank them based on descending order of their scores. In order to score every candidate protein, we employ the weighted degree centrality (*WDC*) [38], relying on the scored disease-specific networks. Specifically, The score of each candidate disease protein, denoted by *SPD*, is computed in terms of the scored interaction between a protein and its direct neighbor. It can be expressed as

3 $$ SPD(u)=\sum^{N_{u}}_{v}W_{u,\;v}  $$

where *N*_*u*_ refers to the set including total neighbors of the protein *u* and *W*_*u*, *v*_ represents the weighted value of edge between the protein *u* and its neighbor *v*. All proteins in the disease-specific networks are ranked in descending order of *SPD*.

## Results and discussions

In this section, we evaluate the ability of PDMG to rank candidate disease genes using the known T2D-gene, subcellular localization and PPI information. The datasets used in the experiments are first described. Next, the diabetes-related networks are discussed. Finally, we analyze the novel diabetes genes predicted by PDMG.

### Data sources

**Known T2D genes.** To form the interaction networks linked to the disease and to detect gene-disease associations from the networks characters, an original set of seed genes known to be associated with the disease is as starting point. We obtain the disease-associated genes of T2D mellitus from OMIM. In OMIM, human genes involved in inherited diseases are recorded in a mini-review format. They are enclosed some information like the gene functions, molecular pathways, and other disease-associated information. To extract a group of T2D-associated genes, we conduct a search of the OMIM database and traverse each OMIM gene record where the term “Diabetes mellitus” is consisted of the “description” field. As a result, 84 OMIM gene records were retrieved. T2D-related entries are shown as Table 1. Based on the HUGO Gene Nomenclature Committee (*HGNC*) database (http://www.genenames.org/), we replace these genes with their corresponding standard symbols and obtain the seed proteins which correspond to these seed genes. We get 27 proteins coded by the known T2D genes, i.e., GPD2, NEUROD1, IRS1, CAPN10, PPARG, SLC2A2, IGF2BP2, WFS1, CDKAL1, HMGA1,ENPP1, GCK, TCF7L2, KCNJ11, ABCC8, MAPK8IP1, UCP3, MTNR1B, HNF1A, TBC1D4, IRS2, LIPC, HNF1B, GCGR, RETN, AKT2 and HNF4A.

**Table 1:** T2D-related gene records

Number	Gene/Locus	Phenotype
1	Gpd2	Diabetes, type 2, susceptibility to
2	Neurod1	Diabetes mellitus, noninsulin-dependent
3	Irs1	Diabetes mellitus, noninsulin-dependent
4	Capn10	Diabetes mellitus, noninsulin-dependent 1
5	Pparg	Diabetes, type 2
6	Slc2a2	Diabetes mellitus, noninsulin-dependent
7	Igf2bp2	Diabetes mellitus, noninsulin-dependent, susceptibility to
8	Wfs1	Diabetes mellitus, noninsulin-dependent, association with
9	Cdkal1	Diabetes mellitus, noninsulin-dependent, susceptibility to
10	Hmga1-rs1,	Diabetes mellitus, noninsulin-dependent,
	Hmga1	susceptibility to
11	Enpp1	Diabetes mellitus, non-insulin-dependent, susceptibility to
12	Gck	Diabetes mellitus, noninsulin-dependent, late onset
13	Pax4	Diabetes mellitus, type 2
14	Slc30a8	Diabetes mellitus, noninsulin-dependent, susceptibility to
15	Tcf7l2	Diabetes mellitus, type 2, susceptibility to
16	Kcnj11	Diabetes mellitus, type 2, susceptibility to
17	Abcc8	Diabetes mellitus, noninsulin-dependent
18	Mapk8ip1	Diabetes mellitus, noninsulin-dependent
19	Ucp3	Obesity, severe, and type II diabetes
20	Mtnr1b	Diabetes mellitus, type 2, susceptibility to
21	Hnf1a	Diabetes mellitus, noninsulin-dependent, 2
22	Pdx1	Diabetes mellitus, type II, susceptibility to
23	Tbc1d4	Diabetes mellitus, noninsulin-dependent, 5
24	Irs2	Diabetes mellitus, noninsulin-dependent
25	Lipc	Diabetes mellitus, noninsulin-dependent
26	Hnf1b	Diabetes mellitus, noninsulin-dependent
27	Gcgr	Diabetes mellitus, noninsulin-dependent
28	Retn	Diabetes mellitus, noninsulin-dependent, susceptibility to
29	Akt2	Diabetes mellitus, type II
30	Hnf4a	Diabetes mellitus, noninsulin-dependent

**Protein subcellular localizations.** The protein subcellular localization data comes from the COMPARTMENTS database [39]. The resource is obtained by integrated a variety of subcellular localization evidences in terms of high-throughput screens, manually curated annotations and sequence-based identification with automatic text mining for all major model organisms. In the COMPARTMENTS database, the different compartments are labeled as: Nucleus, Golgi apparatus, Cytosol, Cytoskeleton, Peroxisome, Lysosome, Endoplasmic reticulum, Mitochondrion, Endosome, Extracellular space and Plasma membrane.

**Protein-protein interactions.** In the experiments, the human protein-protein interactions are downloaded from BioGrid database(Release version BIOGRID-3.2.111) [40]. The human PPINs include 16, 275 proteins and 143, 611 interactions.

### T2D-specific networks

The nearest-neighbor expansion technology is used to construct the T2D-specific protein interaction subnetworks based on the T2D-associated proteins mentioned above and the global PPINs weighted by subcellular localization information. Here, we employ 27 known proteins associated with T2D as the seed diabetes set. The proteins in the weighted PPINs, interacting with the proteins in the seed diabetes set, are pulled out and constitute the candidate T2D protein set. Each interaction between the seed protein and candidate protein composes the diabetes-interaction-set. The two types of proteins (we call them as diabetes-protein-set) and interactions in the diabetes-interaction-set form T2D-specific networks. In the work, the diabetes-protein-set and diabetes-interaction-set contains 445 human proteins and 543 interactions, respectively.

### Novel proteins predicted by PDMG

PDMG is used to calculate the relevance score for each protein in the T2D-specific PPINs. We rank them based on descending order of their scores. Table 2 list top 27 T2D candidate proteins containing 14 known T2D-associated proteins and 13 novel proteins. The 13 novel proteins are not initially retrieved from OMIM database based on the term "diabetes mellitus". The results show that our prioritizing technology demonstrates very high specificity: out of 27 top-ranking proteins, 14 proteins are known T2D-related proteins in terms of OMIM annotation. Meanwhile, it can be found that the scores of all known proteins but two ones (HNF1B and GCK) are larger than those of other candidate proteins. Furthermore, to examine PDMG’s ability to predict novel diabetes-associated proteins, we use literature study method to determine if the predicted proteins are associated with diabetes. The retrieve results display that out of 13 novel proteins, 8 proteins have been proved to be diabetes-related proteins by literature in the PubMed database (http://www.ncbi.nlm.nih.gov/pubmed). The 8 novel proteins are presented as follows.

**Table 2:** Top 27 rank-ordered T2D relevant proteins

Rank	Protein	Score	Description	Diabetes relevance
1	PPARG	85.83	peroxisome proliferator activated	Known
			receptor gamma, T2D, susceptibility to	
2	HMGA1	63.99	high mobility group AT-hook 1, Diabetes,	Known
			noninsulin-dependent, susceptibility to	
3	HNF4A	60.08	hepatocyte nuclear factor 4 alpha,	Known
			Diabetes mellitus, noninsulin-dependent	
4	IRS1	45.12	insulin receptor substrate 1, Diabetes,	Known
			noninsulin-dependent	
5	HNF1A	24.21	HNF1 homeobox A, Diabetes,	Known
			noninsulin-dependent, 2	
6	AKT2	23.28	v-akt murine thymoma viral	Known
			oncogene homolog 2, Diabetes, type II	
7	TCF7L2	20.03	transcription factor 7 like 2,	Known
			Diabetes, type 2, susceptibility to	
8	IGF2BP2	17.77	insulin like growth factor 2	Known
			mRNA binding protein 2, Diabetes,	
			noninsulin-dependent, susceptibility to	
9	MAPK8IP1	14.52	mitogen-activated protein kinase 8	Known
			interacting protein 1, Diabetes,	
			noninsulin-dependent	
10	IRS2	12.78	insulin receptor substrate 2,	Known
			Diabetes, noninsulin-dependent	
11	NEUROD1	7.03	neurogenic differentiation 1,	Known
			Diabetes, noninsulin-dependent	
12	UBC	6.52	ubiquitin C	Novel
13	HNF1B	6	HNF1 homeobox B, Diabetes,	Known
			noninsulin-dependent	
14	EP300	4	E1A binding protein p300	Novel
15	CREBBP	4	CREB binding protein	Novel
16	ESR1	3.47	estrogen receptor 1	Novel
17	AKT1	3.02	v-akt murine thymoma	Novel
			viral oncogene homolog 1	
18	NRF1	3.02	NFKB repressing factor	Novel
19	PCBD1	3	pterin-4 alpha-carbinolamine dehydratase 1	Novel
20	SP1	3	Sp1 transcription factor	Novel
21	HDAC4	3	histone deacetylase 4	Novel
22	YWHAB	2.49	tyrosine 3-monooxygenase/tryptophan	Novel
			5-monooxygenase activation protein beta	
23	EGFR	2.47	epidermal growth factor receptor	Novel
24	GCK	2.45	glucokinase, Diabetes, noninsulin-dependent,	Known
			late onset	
25	ELAVL1	2.45	ELAV like RNA binding protein 1	Novel
26	APP	2.29	amyloid beta precursor protein	Novel
27	SUMO2	2.01	small ubiquitin-like modifier 2	Novel

#### CREBBP #15

Rende et al. find that CREB binding protein (CREBBP) plays suggestive roles in linking Type 2 diabetes [41]. Their study reveals that heterozygous CREBBP defect leads to raised effects of hormones like leptin and adiponectin, insulin resistance and preventing obesity. Manabe et al. observe that the mRNA expression of CREBBP is reduced in the uteri of ovariectomized STZ-treated diabetic mice [42]. A recent literature report [43] shows that, compared with healthy conditions, the expressing of histone acetyltransferases CREBBP in latent autoimmune diabetes in adults patients is downregulated.

#### ESR1 #16

Linner et al. conclude that the rs2207396 mutation in ESR1 suggests the risk of type 2 diabetes in hypogonadal men [44]. By investigating the relationship between single nucleotide polymorphisms (SNPs) of the candidate gene and the quantitative traits related to metabolic syndrome in Han Chinese type 2 diabetes, Wei et al. [45] find that Rs722208 of ESR1 is associated with fasting plasma glucose (FPG)(*P*= 0.045).

#### AKT1 #17

Devaney et al. [46] report that AKT1 is a risk factor for metabolic syndrome and insulin resistance which is one of the five essential endophenotypes linked to T2D. Hami et al. find a significant bilateral downregulation of AKT1 gene expression in the hippocampus of pups born to diabetic mothers [47].

#### NRF1 #18

By researching defect of Nuclear factor-erythroid 2-related Factor 1 (NRF1) in beta-cells, Zheng et al. discover that Nrf1 acts as an essential regulator of mitochondrial function, glucose metabolism and insulin secretion [48]. Specifically, Nrf1 inactivation in beta-cells results in a pre-T2D phenotype because of impairment of insulin secretion and disruption of glucose metabolism [48]. In the study from Hirotsu et al., Nrf1 over-expression has a negative impact on both glucose utilization and production in the liver by suppressing the genes related to both glycolysis and gluconeogenesis [49].

#### PCBD1 #19

The findings from Ferre et al. suggest that a PCBD1 deficiency may cause hypomagnesemia and diabetes [50]. Simaite et al. observe an abundant expression of Pcbd1 in the developing pancreas of both mouse and Xenopus embryos [51]. The genetic evidence obtained by them displays that PCBD1 variations can lead to early-onset nonautoimmune diabetes with characteristics like dominantly inherited HNF1A-diabetes.

#### YWHAB #22

YWHAB interacts with GCGR, a type 2 diabetes-related protein. To examine the effect of YWHAB on GCGR function, Han et al. investigate glucose production in primary mouse hepatocytes. They discover that YWHAB is overexpressed in mouse hepatocytes. In other words, YWHAB inhibits glucose production [52]. Studies show that YWHAB may plays a critical role in glucose metabolism. YWHAB actually regulates the activity of ChREBP (glucose responsive transcription factor), carbohydrate response element-binding protein, which has important influence on the glucose-mediated induction of proteins associated with hepatic glycolysis and lipogenesis [53]. Besides, YWHAB also controls the activity of AKT, which mediates insulin signaling [54].

#### EGFR #23

Chen et al. suggest that EGFR (epidermal growth factor receptor) mediates TGF-b-induced renal fibrosis and is inhibited by the EGFR inhibitor, erlotinib, in STZ-induced diabetic mice [55]. More recently, they also report the resistance of podocyte-specific EGFR knockout mice to the development of diabetes-associated podocyte damage [56].

#### SUMO2 #27

The transcriptional activity of T and B cells is negatively regulated by the mouse SUMO2 [57, 58]. The mouse SUMO2 in T cells is overexpressed, which inhibits the production of both Th1 and Th2 cytokines [57, 58]. This means that the mouse SUMO2 plays a more complex role in the progression of autoimmune diabetes. The early literature [59] also shows that SUMO is related to NF-kB activation and may thus be linked to type 1 diabetes with apoptosis in pancreatic beta cells.

## Conclusions

With the available PPI data increasing rapidly, a unprecedented opportunity for predicting disease-associated genes/proteins at the network level is appear. The PPINs have been widely adopted by many state of the art algorithms to address the gene prioritization problem. They are based on the principle that the genes/proteins causing similar diseases tend to cluster together in the network. However, the high false positive rates and false negative rates of the available PPI data have a negative influence on the accuracy of methods identifying disease genes/proteins only by the topological properties of the networks. To improve the prediction, researchers develop all kinds of new approaches to predict candidate disease genes via combining other data from different sources with PPINs. But these methods neglect an obvious fact proteins don’t perform their desired functions unless they are localized at the appropriate subcellular compartments. In this work, subcellular localization data are integrated with PPINs. The combination is achieved by building disease-specific PPINs and employing them in the prioritization. Specifically, OMIM is used to obtain seed genes/proteins of type 2 diabetes. With these seed proteins, we produce T2D-specific PPINs from the weighted PPINs based on the nearest-neighbor expansion approach. And then the scores of candidate T2D proteins are calculated by WDC method. Finally, we rank the proteins based on descending order of their scores.

In order to prove PDMG’s ability to predict potential disease-related proteins, we employ the literature review method to analyze the novel proteins/genes predicted by PDMG. The results show that PDMG has predicted 13 novel proteins in top 27 candidate proteins. Out of the 13 novel proteins, 8 proteins CREBBP, ESR1, AKT1, NRF1, PCBD1, YWHAB, EGFR, SUMO2 are associated with diabetes in literature. The evidences display that the 8 novel proteins are recovered from the interaction data and subcellular localization information analysis although they are not retrieved from OMIM database. Therefore, PDMG method can make up for the false negatives (to an extent) of PPINs. Besides, according to the ranked candidate proteins, one may gain many new biological suppositions about the new protein functions in the context of protein interaction networks out of scope of this work.
